# Lipid-lowering drugs and inflammatory bowel disease’s risk: a drug-target Mendelian randomization study

**DOI:** 10.1186/s13098-023-01252-1

**Published:** 2024-01-09

**Authors:** Jiaxi Zhao, Rong Chen, Mengqi Luo, Hongping Gong, Kaixin Li, Qian Zhao

**Affiliations:** 1 General Practice Ward/International Medical Center Ward General Practice Medical Center, West China Hospital, Sichuan University, Chengdu, 610041 Sichuan China; 2 Department of Rehabilitation Medicine, The First Affiliated Hospital, Sun Yat-Sen University, Guangzhou, Guangdong China; 3 Department of Gastroenterology and Hepatology, West China Hospital, Sichuan University, Chengdu, Sichuan China; 4 Department of Nephrology, Huadong Hospital, Shanghai, China

**Keywords:** Inflammatory bowel disease, Lipid- lowering drug, Mendelian randomization, Crohn’s disease, Ulcerative colitis

## Abstract

**Background:**

Inflammatory bowel disease (IBD) has been associated with lipid-lowering drugs in observational studies. Drug-target Mendelian randomization (MR) was utilized in this study to examine the causal relationship between lipid-lowering drugs and incidence of IBD, aiming to identify new preventive uses for the drugs.

**Methods:**

We identified instrumental variables for three classes of lipid-lowering drugs: HMGCR inhibitors, PCSK9 inhibitors, and NPC1L1 inhibitors, using data from the Global Lipids Genetics Consortium. Summary statistics of IBD were obtained from UK Inflammatory Bowel Disease Genetics. The summary-data-based MR (SMR) and the inverse-variance weighted (IVW) MR were used for analysis. Sensitivity analyses were performed by conventional MR methods.

**Results:**

The SMR analysis showed no significant genetic association between increased gene expression of HMGCR, PCSK9, and NPC1L1 and IBD, Crohn’s disease (CD) and ulcerative colitis (UC). According to IVW-MR analysis, increased HMGCR expression is associated with a reduced risk of IBD (OR = 0.73, 95% confidence interval (CI) 0.59–0.90, P = 0.003) and CD (OR = 0.75, 95% CI 0.57–0.97, P = 0.03), but not with UC. Additionally, increased NPC1L1 gene expression was associated with elevated risk of IBD (OR = 1.60, 95% CI 1.07–2.40, P = 0.023), but not with CD and UC. However, no significant causal relationships were found between PCSK9 gene expression and IBD, CD, and UC. The sensitivity analysis demonstrated no evidence of heterogeneity or pleiotropy among the reported results.

**Conclusions:**

The heightened expression of genetic variations in HMGCR inhibitor targets could potentially reduce the risk of IBD and CD, while genetic variation in the expression of NPC1L1 targets was positively associated with IBD.

**Supplementary Information:**

The online version contains supplementary material available at 10.1186/s13098-023-01252-1.

## Key messages

What is already known? IBD were associated with lipid-lowering drugs in previous studies, but the causal relationship is unknown. There were two studies exploring the relationship, but the results were inconsistant.

What is new here? We used two methods to investigate the relationship between lipid-lowering drugs and IBD and obtained different results compared to previous studies.

How can this study help patient care? This study provides new possibilities for the future use of existing lipid-lowering drugs in the treatment of IBD, representing a case of “repurposing old drugs.”

**Supplementary Information:**

The online version contains supplementary material available at 10.1186/s13098-023-01252-1.

## Introduction

Inflammatory bowel disease (IBD) is a chronic disorder characterized by inflammation in the gastrointestinal tract [1], comprising two main conditions: Crohn’s disease (CD) and ulcerative colitis (UC) [2, 3]. It affects millions of individuals worldwide, imposing a substantial burden in terms of morbidity and healthcare costs [4, 5]. Current therapies primarily focus on managing symptoms and reducing inflammation, however, their efficacy varies among patients and some individuals may experience side effects or develop resistance to these treatments over time [6, 7]. Finding a drug that reduces the risk of IBD would be highly beneficial. The lipid-lowering drugs, commonly used to manage dyslipidemia and reduce cardiovascular risk [8, 9], have shown promise in both preclinical and clinical studies on other diseases due to their anti-inflammatory properties [10, 11]. Recently, there has been a growing interest in investigating the potential of lipid-lowering drugs as therapeutic targets for IBD [12, 13]. Previous research evidence suggested that statins, as inhibitors of 3-hydroxy-3-methylglutaryl coenzyme A reductase (HMGCR), might reduce the risk of CD [14] and had an adjunctive role in the treatment of UC [15]. The rationale behind it lies in the intricate connection between lipid metabolism and inflammation, as dysregulated lipid homeostasis has been implicated in the pathogenesis of IBD [12, 13]. However, the therapeutic efficacy of statins in IBD is in dispute, with studies finding that statins do not prevent new-onset IBD [16] and are not associated with beneficial effects in patients with UC [17]. Except for statins, there are two commonly used lipid-lowering medications that target specific factors: ezetimibe targeting Niemann-Pick C1-like 1 (NPC1L1) [18] and proprotein convertase subtilisin/kexin type 9 (PCSK9) inhibitors [19], research on their associations with IBD are limited.

The contradictory results from these investigations underscore the importance of conducting additional research on the impact of different lipid-lowering medications in the context of IBD. Nevertheless, observational studies are subject to inherent limitations, such as residual confounding and reverse causation [20, 21]. Therefore, the Mendelian randomization (MR) has been chosen as a robust method for this study.

MR is a powerful method that uses genetic variants as instrumental variables to investigate causal relationships between exposures and outcomes [22]. It takes advantage of the random allocation of genetic variants during meiosis, which makes them less susceptible to confounding and reverse causation compared to traditional observational studies [23]. In MR analysis, by examining the association between these instrumental variables and the outcome of interest, researchers can infer a causal relationship between the exposure and the outcome [21].In this study, by targeting specific pathways involved in lipid metabolism, such as the drug-target MR analysis of lipid-related genes [24], we aimed to uncover the potential association between lipid-lowering drugs and incidence of IBD [25–27].

## Materials and methods

### Study design

**Fig. 1:**
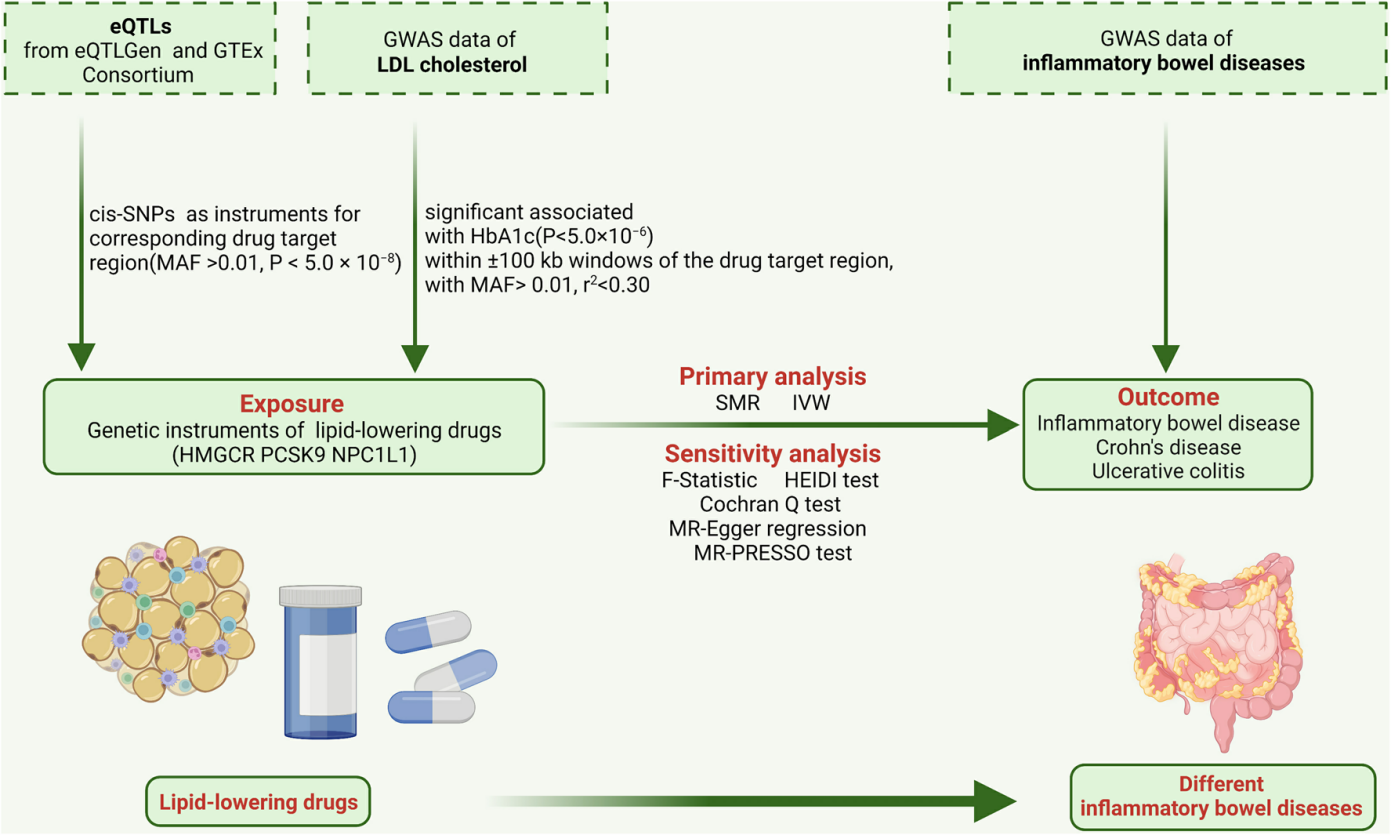
The flowchart of the study

The current MR study samples consisted of data of publicly accessible genome-wide association studies (GWAS) and expression quantitative trait loci (eQTL) studies. This study primarily employed two analytical methods, the summary-data-based MR (SMR) and the inverse-variance weighted (IVW) MR. For detailed information about the data sources were shown in Additional file 1: Table S1. The flowchart is depicted in Fig. 1.

All participants involved in the included studies had provided informed consent, and the respective institutional review boards had granted the necessary approvals. No further approvals were necessary for the current study.

### Selection of genetic instruments

We included three classes of lipid-lowering drugs as exposures: HMGCR inhibitors, PCSK9 inhibitors, and NPC1L1 inhibitors, as shown in Table 1. We used eQTLs for the available target genes of these drugs (i.e., HMGCR, PCSK9, and NPC1L1) as proxies for exposure to each lipid-lowering drug. The summary-level data of eQTLs were obtained from the eQTLGen Consortium [28] (https://www.eqtlgen.org/) or the GTEx Consortium V8 [29] (https://gtexportal.org/), and the detailed information was presented in Table 1 and Additional file 1: Table S1. We identified significant single-nucleotide polymorphisms (SNPs) associated with HMGCR or PCSK9 expression in blood by setting a significance threshold (P < 5.0 × 10^−8^) and minor allele frequency (MAF) > 1%. However, no significant eQTL for NPC1L1 was found in blood or other tissues, so NPC1L1 SNPs were obtained from subcutaneous adipose tissue. In this study, only cis-eQTL within a 1 Mb range on the coding gene's one side were included as genetic instruments. In blood, the genes HMGCR and PCSK9 exhibited 921 and 24 positive eQTLs, respectively. And in adipose subcutaneous tissue, the NPC1L1 gene demonstrated 11 positive eQTLs, as shown in Table 2 and Additional file 1: Table S2.

**Table 1:** Summary information of lipid-lowering drug categories, targets, and encoding genes

Drug categories	Drug targets	Encoding genes	Gene region(in GRCh37 from Ensembl)
HMGCR inhibitors	3-hydroxy-3-methylglutaryl coenzyme A reductase	HMGCR	Chromosome 5: 74,632,154-74,657,929
PCSK9 inhibitors	proprotein convertase subtilisin/kexin type 9	PCSK9	Chromosome 1: 55,505,221-55,530,525
NPC1L1 inhibitors	Niemann-Pick C1-Like 1	NPC1L1	Chromosome 7: 44,552,134-44,580,914

**Table 2:** Information of genetic instruments

Exposure	Genetic instruments	
	Genetic variants associated with eQTL (MAF > 1%; *P* < 5.0 × 10^−8^)	Genetic variants associated with LDL-C levels (MAF > 1%; r ^2^ < 0.30), associated with LDL-C (*P* < 5.0 × 10^−6^)
HMGCR	921 common cis-eQTLs top SNP: rs6453133	7 common SNPs
PCSK9	24 common cis-eQTLs top SNP: rs472495	12 common SNPs
NPC1L1	11 common cis-eQTLs top SNP: rs41279633	4 common SNPs

Apart from employing eQTLs as instruments, we conducted an assessment of the relationship between each genetic variant and LDL cholesterol (LDL-C) levels to determine the potential lipid-lowering effects achieved by inhibiting drug targets. The Global Lipids Genetics Consortium (GLGC) [30] served as our data source for LDL-C, comprising sample size of 173,082 individuals. We carefully selected SNPs as genetic instruments based on specific criteria: exhibited low linkage disequilibrium (r^2^ < 0.30), possessed a MAF > 1%, and demonstrated a significant association with LDL-C levels both within a 100 kb region surrounding the drug target (P < 5.0 × 10^−6^). (Information of genetic instrumental variants associated with LDL cholesterol located within 100 kb windows from gene HMGCR, PCSK9, or NPC1L1 and inflammatory bowel disease were shown in Additional file 1: Table S4-S6.

### Outcome sources

The outcome data for this study was derived from the UK Inflammatory Bowel Disease Genetics [31], encompassing IBD, CD, and UC. We have 25,042 cases and 34,915 controls for IBD, 12,194 cases and 28,072 controls for CD, and 12,366 cases and 33,609 controls for UC. (Note: As IBD mainly includes CD and UC, this study investigated IBD, CD, and UC collectively as outcomes. Please take note of the entities referred to by the term IBD.)

### Statistical analysis

#### Primary MR analysis

The SMR method-based summary data was employed using eQTLs as tools to investigate the association between gene expression levels and the interested outcome using summary data from GWAS and eQTL studies. The SMR software version 1.03 (https://cnsgenomics.com/software/smr/#Overview) was utilized. Additionally, when using genetic variants associated with LDL-C levels as genetic instruments, IVW-MR method was employed for effect estimation. The analysis was conducted using the TwoSampleMR package in R software version 4.2.2.

#### Sensitivity analysis

We used the F-statistic as a measure of the strength of the SNP for evaluating the instrument in our study [32]. An F-statistic greater than 10 was used to minimize weak instrument bias. For the SMR method, we employed the the heterogeneity in dependent instruments (HEIDI) test to assess whether the observed association between gene expression and outcomes was due to the linkage disequilibrium [33]. And a P-value less than 0.01 indicated that the association might be due to linkage disequilibrium. The presence of horizontal pleiotropy suggested that a SNP might be associated with the expression of multiple genes. We identified other genes within a 1 Mb window and performed SMR analysis to examine whether the expression of these genes was related to the outcome.

For the IVW-MR method, we utilized multiple approaches for sensitivity analysis. The Cochran's Q test was employed to test for heterogeneity, with a P-value less than 0.05 indicating the presence of heterogeneity. The MR Egger regression and Mendelian Randomization Pleiotropy RESidual Sum and Outlier (MR-PRESSO) analysis were used to assess potential horizontal pleiotropy of the SNP [32]. In the MR Egger regression, the intercept term served as a useful indicator of directional horizontal pleiotropy, where P < 0.05 suggested the presence of horizontal pleiotropy. The MR-PRESSO analysis could identify outliers due to horizontal pleiotropy and provide adjusted estimates. All of these analyses were implemented in R software version 4.2.2.

## Results

### Primary analysis

**Fig. 2:**
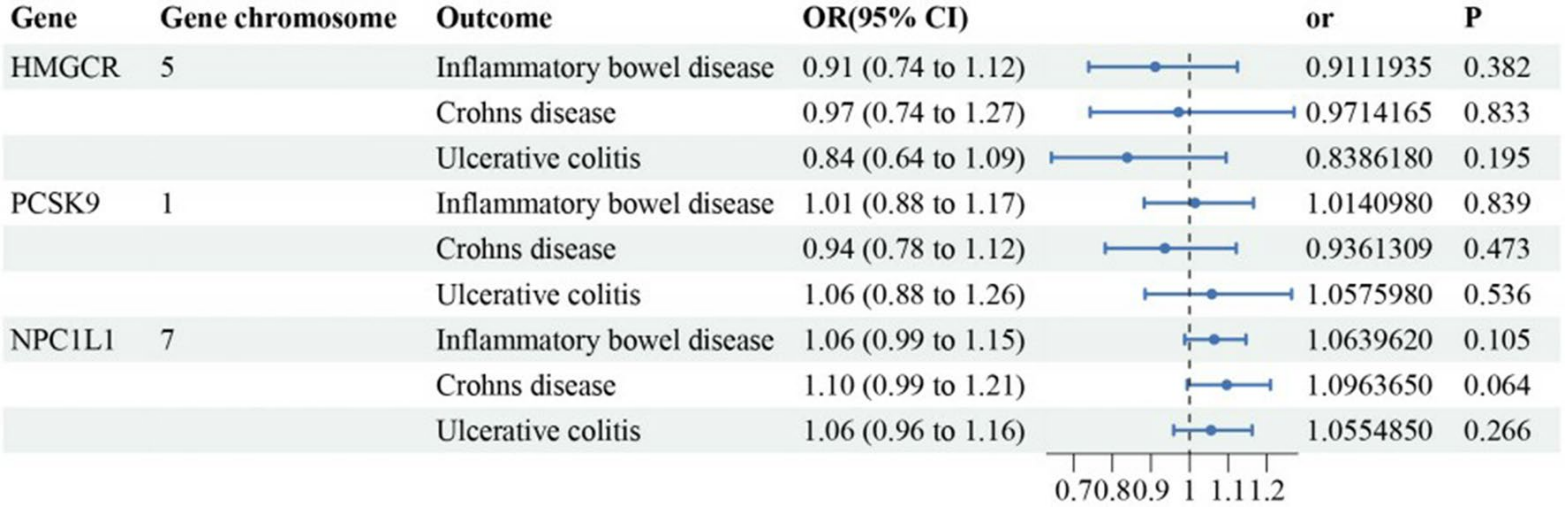
The results of the SMR analysis

According to the SMR analysis results from Fig. 2 and Additional file 1: Table S2, there were no significant genetic associations between the increased gene expression of HMGCR, PCSK9, and NPC1L1 and IBD, CD, and UC (SMR method: all P > 0.05).

**Fig. 3:**
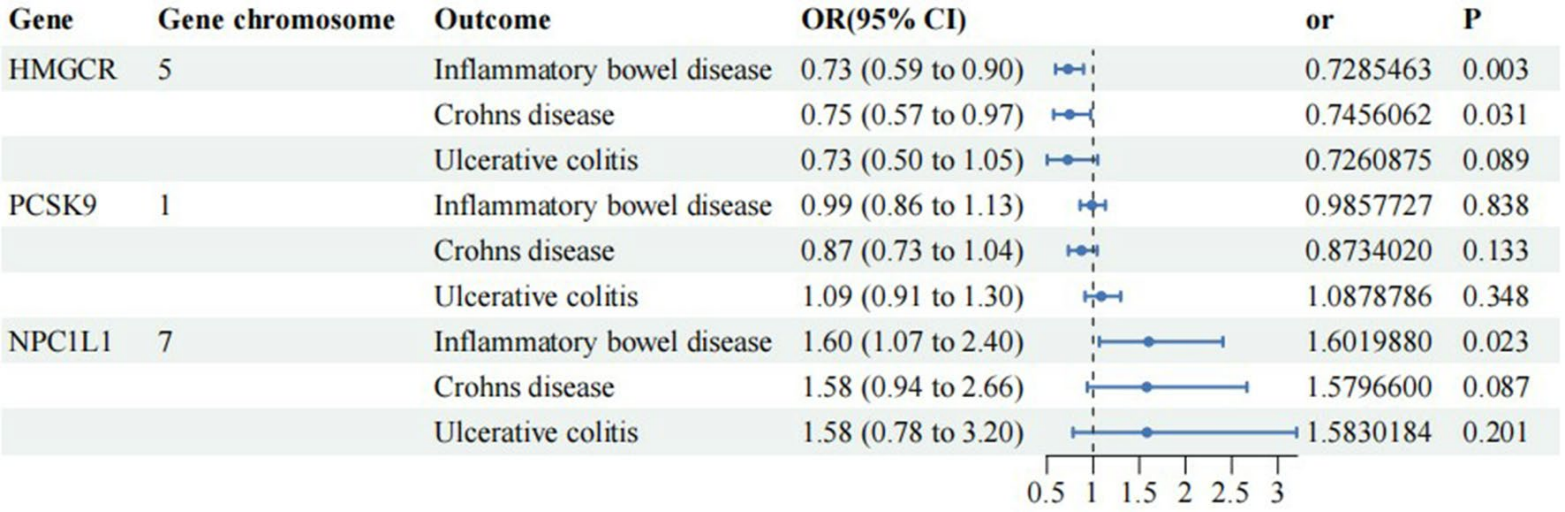
The results of IVW-MR analysis

The IVW-MR analysis results presented in Fig. 3 and Additional file 1: Table S3 indicated a significant correlation between increased gene expression of HMGCR and a reduced risk of IBD and CD. The odds ratio (OR) for IBD was 0.73, with a 95% confidence interval (CI) of 0.59–0.90 (P = 0.003), while the OR for CD was 0.75, with a 95% CI of 0.57–0.97 (P = 0.03). Additionally, there was an association between increased NPC1L1 gene expression and an elevated risk of IBD (P = 0.023, OR = 1.60, 95% CI: 1.07–2.40). However, no significant causal relationships were found between HMGCR gene expression and UC, between PCSK9 gene expression and IBD, CD, and UC, and between NPC1L1 gene expression and CD and UC (IVW-MR method: P > 0.05).

### Sensitivity analysis

The SMR analysis results, as confirmed by the HEIDI test, indicated that the observed associations were not attributed to linkage disequilibrium (P > 0.01; Additional file 1: Table S2). According to the Cochran Q test, the IVW-MR analysis results demonstrated no evidence of heterogeneity among the reported results (all P > 0.05; Additional file 1: Table S3). Furthermore, both the MR Egger regression and MR-PRESSO analysis results provided evidence that there was no significant horizontal pleiotropy (all P > 0.05; Additional file 1: Table S3).

## Discussion

To our knowledge, this study represented a pioneering endeavor in utilizing drug-target MR and SMR analyses to investigate the influence of lipid-lowering drug targets on the susceptibility to IBD, encompassing both CD and UC. Notably, our findings revealed a positive association between elevated expression of the HMGCR gene and a decreased risk of IBD and CD, although no such correlation was observed with UC. Conversely, increased expression of the NPC1L1 gene was linked to an elevated risk of IBD, while no significant associations were observed in CD and UC. Furthermore, no significant causal relationships were identified between PCSK9 gene expression and the occurrence of IBD, CD, and UC.

In our study, we observed a positive correlation between increased HMGCR gene expression and a reduced risk of IBD and CD, but not in UC. These findings suggested that higher gene expression levels of HMGCR, a key enzyme involved in cholesterol synthesis, may have a protective effect against the development of IBD and CD. The findings were align with earlier research suggesting a correlation between the use of lipid-lowering medications that target HMGCR, such as statins, have been associated with a reduced risk of IBD [34]. Moreover, statin use was subsequently found to decrease disease activity and downregulate inflammatory markers in patients with rheumatoid arthritis by a meta-analysis of 15 RCTs [35]. Previous studies found that statins as competitive inhibitors of HMG-CoA reductase, leading to a decrease in intracellular cholesterol levels, which in turn, upregulates LDL receptors on the membranes of liver cells, facilitating the clearance of circulating LDL-C particles [36, 37]. In addition to their lipid-lowering properties, statins also possess anti-inflammatory abilities [38, 39]. The results of this study were supported by previous research, indicating that alterations in the expression levels of HMGCR inhibitors drug targets may negatively contribute to the pathogenesis of IBD. As one of the primary diseases within IBD, UC exhibited no significant statistical association with targeted expression of the HMGCR gene. This lack of association may be attributed to the difference of genetic characteristics between CD and UC. However, it is important to note that this study solely investigates the causal relationship between drugs and diseases from a genetic perspective. In the real world, numerous factors may contribute to the correlation between these two.

In addition to our findings on the association between HMGCR, and IBD, we also observed an interesting relationship between the NPC1L1 gene and the risk of IBD. The NPC1L1 protein is known for its role in the absorption of dietary cholesterol, and genetic variations in the NPC1L1 gene have been shown to impact cholesterol absorption and serum cholesterol levels [40, 41]. The representative medication of NPC1L1 inhibitors is ezetimibe [42]. In a previous study [43], researchers investigated the genotyping of the NPC1L1 gene in both healthy individuals and patients with hepatitis C virus and IBD. The study found statistically significant associations between the 1735GG variant of the NPC1L1 gene and total cholesterol (TC) levels in patients with CD, which suggested that the NPC1L1 1735GG variant might be associated with higher TC levels in CD patients, indicating a potential link between this genetic variant and malnutrition in CD. These findings highlighted the importance of considering the role of cholesterol metabolism and absorption in the development and progression of IBD. Further researches were needed to elucidate the underlying mechanisms linking NPC1L1 gene variations, cholesterol levels, and the pathogenesis of IBD. Understanding these relationships may provide novel insights into the potential therapeutic targets for managing IBD and its associated complications. It is important to note that the absence of significant causal relationships between PCSK9 gene expression and the occurrence of IBD, CD, and UC in the present study. However, in recent study, the PCSK9 has been recognized as a biomarker for inflammation [44, 45], which indicates the potential involvement of PCSK9 in the process of intestinal inflammation. Futhermore, an observational study has shown that during active phases of UC, serum levels of PCSK9 increase [46], but our study didn’t show a potential association between PCSK9 and the development and severity of UC, additional studies are required to validate this correlation.

Recently, two MR studies have conducted analyses on the impact of lipid-lowering medications on IBD. In one study, it was found that inhibition of the lipid-lowering drug target NPC1L1 gene expression may increase the risk of developing IBD, which aligns with our research findings [47]. Another study, utilizing drug-targeted MR analysis, indicated an association between the expression of PCSK9 and an elevated risk of IBD, CD, and UC [13]. However, our study did not uncover similar results. In comparison to these two studies, our approach is more scientifically reliable, employing both SMR and IVW-MR methodology, despite the disparities in their results. Furthermore, our research revealed that increased genetic expression in HMGCR inhibitor targets (gene: HMGCR) may lower the risk of IBD and CD. Several factors could lead to heterogeneity between our analysis and previous studies. Firstly, it is important to consider the complexity of the genetic architecture underlying these conditions. UC, CD, and IBD are multifactorial diseases, influenced by the interplay of various genetic, environmental, and lifestyle factors [48–50]. Secondly, the genetic contribution to these diseases is likely polygenic, involving multiple genes with small effect sizes. Therefore, it is possible that the individual effects of HMGCR, PCSK9, and NPC1L1 gene expressions on UC, CD, and IBD risk are too small to be detected in our study.

This study possesses several advantages. Firstly, it employs the medication-targeted MR method to systematically investigate the causal relationship between lipid-lowering medications and IBD. Secondly, building upon previous MR studies on lipid-lowering medications and IBD, this research yields novel findings, providing valuable insights for reconsidering the application of lipid-lowering medications in future IBD prevention and treatment strategies. Additionally, the exposure and outcome data utilized in this study are based on large-scale GWAS data, thereby enhancing its scientific reliability. However, this study also has certain limitations. Firstly, the predominantly European variable data restricts the applicability to other populations. Future research could consider diverse ethnic groups like Asians for validation. Secondly, while both SMR and IVW-MR methods were used, only IVW-MR found significant associations, possibly due to multifactorial factors. Additionally, like other MR studies, this research solely focuses on the occurrence risk of IBD as an endpoint, without delving into the disease progression, severity, and complications. With the emergence of relevant GWAS dataset, further exploration of these aspects can be pursued in the future.

In conclusion, the increased genetic expression in HMGCR inhibitor targets (gene: HMGCR) may lower the risk of IBD and CD, whereas genetic variation in NPC1L1 targets (gene: NPC1L1) expression showed a positive correlation with IBD. Further research with larger sample sizes, comprehensive genetic coverage, and consideration of other potential confounding factors is warranted to fully understand the potential role of these lipid-lowering drugs targeted genes in the development and progression of IBD, CD, and UC.

### Supplementary Information

**Additional file 1: Table S1.** Information of eQTL and GWAS summary data. **Table S2.** SMR association between expression of gene HMGCR, PCSK9, or NPC1L1 and inflammatory bowel diseases. **Table S3.** IVW-MR association between LDL cholesterol mediated by gene HMGCR, PCSK9, or NPC1L1 and inflammatory bowel diseases. **Table S4.** Information of genetic instrumental variants associated with LDL cholesterol located within 100 kb windows from gene HMGCR, PCSK9, or NPC1L1 and inflammatory bowel disease. **Table S5.** Information of genetic instrumental variants associated with LDL cholesterol located within 100 kb windows from gene HMGCR, PCSK9, or NPC1L1 and Crohn’s disease. **Table S6.** Information of genetic instrumental variants associated with LDL cholesterol located within 100 kb windows from gene HMGCR, PCSK9, or NPC1L1 and ulcerative colitis.

## Data Availability

Data available on request from the authors. The data that support the findings of this study are available from the corresponding author. Readers can obtain information by requesting data from corresponding authors.

## Abbreviations

CD: Crohn’s disease
CI: Confidence interval
eQTL: Expression quantitative trait loci
GWAS: Genome-wide association studies
GLGC: Global Lipids Genetics Consortium
HEIDI: Heterogeneity in dependent instruments
HMGCR: 3-Hydroxy-3-methylglutaryl coenzyme A reductase
IBD: Inflammatory bowel disease
IVW: Inverse-variance weighted
LDL: Low-density lipoprotein
LDL-C: LDL cholesterol
MR: Mendelian randomization
MAF: Minor allele frequency
MR-PRESSO: Mendelian Randomization Pleiotropy RESidual Sum and Outlier
NPC1L1: Niemann-Pick C1-like 1
OR: Odds ratio
PCSK9: Proprotein convertase subtilisin/kexin type 9
SMR: Summary-data-based MR; UC: ulcerative colitis
